# Vaccine Adjuvants: Mode of Action

**DOI:** 10.3389/fimmu.2013.00214

**Published:** 2013-07-31

**Authors:** Ennio De Gregorio, Elena Caproni, Jeffrey B. Ulmer

**Affiliations:** ^1^Novartis Vaccines and Diagnostics Inc., Siena, Italy; ^2^Novartis Vaccines and Diagnostics Inc., Cambridge, MA, USA

**Keywords:** adjuvant, vaccine, human, innate immunity, toll-like receptor

## Abstract

Vaccines were first introduced more than 200 years ago and have since played a key role in the reduction of morbidity and mortality caused by infectious diseases. Many of the safest and most effective vaccines in use today are based on attenuated live viruses, as they mimic a live infection without causing disease. However, it is not always practical to take this approach, such as when it may not be safe to do so (e.g., for viruses that cause chronic infections such as HIV) or may not be feasible to manufacture (e.g., for viruses that do not grow well in cell culture such as HCV). In addition, it may preferable in some cases to target immune responses toward specific antigens from the pathogen, rather than the entirety of the genome. In these cases, subunit vaccines consisting of antigens purified from the pathogen or produced by recombinant DNA technology are being developed. However, highly purified proteins are typically not inherently immunogenic, as they usually lack the means to directly stimulate the innate immune system, and often require the addition of adjuvants to enhance vaccine potency. Despite more than a century of human use, only a few adjuvants are licensed today. However many adjuvants have been tested in humans and are in advanced stages of development. Much of the early work on adjuvants discovery and development was empirical producing safe and effective products, but without a clear understanding of how they worked. Recent insight into the functioning of the innate immune system has demonstrated its important role in triggering and shaping the adaptive immune response to vaccines.

## Introduction

Adjuvants have been used in human vaccines for almost a century, yet very few adjuvants are licensed for human use. This has been due, in part, to a lack of understanding of their mechanism of action. However, recent insights into the innate immune system and its importance in initiating the adaptive immune response have sparked the rational design and development the next generation of adjuvants. Several studies have validated one class of pattern recognition receptors (PRRs) called Toll-like Receptors (TLRs) as vaccine adjuvant targets. Various TLR agonists have been tested in humans and the TLR4 agonist monophosphoryl-lipid A (MPL) has been recently licensed in Europe and the USA for a vaccine that prevents human papilloma virus (HPV) infection (Table 1). This chapter will focus on both well established and exploratory adjuvants to provide an overview of our current understanding of vaccine adjuvant mechanism of action and how this information may be used in the discovery of the next generation of products.

**Table 1:** **Adjuvants evaluated in humans**.

Adjuvants	Class	Vaccine
**TLR-DEPENDENT ADJUVANTS**		
**AS04**	Alum-adsorbed TLR4 agonist (31)	HBV, HPV
**RC-529**		HBV
**CpG 7909**	TLR9 agonist (39)	HBV, Influenza, etc.
**CpG1018**		HBV, Cancer
**IC31**		TB
**Imiquimod**	TLR7 agonist (43)	Cancer
**Flagellin**	TLR5 agonist (42)	Influenza
**AS01**	Combo TLR4	Malaria
**AS02**	Combo TLR4	Malaria, TB, Cancer
**AS15**	TLR4 + TLR9	Cancer
**TLR-INDEPENDENT ADJUVANTS**		
**Alum**	Mineral salts (1), (2)	Diphtheria, tetanus, pneumococcus, etc.
**MF59**	Oil-in-water emulsion (22), (29)	Influenza
**AS03**		Influenza
**AF03**		influenza
**Virosomes**	Liposomes	HAV
**Iscomatrix**	Combination	HCV, influenza, HPV, cancer
**Montanide ISA51**	Oil-in-water emulsion	Malaria, HIV, cancer
**Montanide ISA720**		Malaria, HIV, cancer
**LT**	Bacterial toxins	Influenza, ETEC
**LTK63**		Influenza, TB, HIV

*TLR-dependent and TLR-independent adjuvants have been tested in human clinical trials. Those shown in green are components of licensed human vaccines, while those in orange have been tested in clinical trials, but are not yet approved. References cited are provided for those adjuvants discussed in detail in the text.ETEC, enterotoxigenic *E. coli*; HAV, hepatitis A virus; HBV, hepatitis B virus, HCV, hepatitis C virus; HIV, human immunodeficiency virus; HPV, human papilloma virus; LT, labile toxin; TB, tuberculosis*.

## Mode of Action of Aluminum Salts

Aluminum salts (aka alum) have been in wide use with human vaccines for almost a century, with the first proof of concept studies in animal models published in 1926 (1). This class of adjuvants, which includes aluminum phosphate, aluminum hydroxide, and aluminum hydroxyphosphate, is a component of various viral and bacterial vaccines such as diphtheria, tetanus, pertussis, hepatitis A and B, rabies, anthrax, and others. Alum formulations are particulate in nature, to which the vaccine antigens are adsorbed, albeit with distinct characteristics among the different forms of alum salts (2). This adsorption can result in increased antigen stability *in vitro* (3) and led to the initial assumption that alum creates a depot *in situ*, thereby allowing slow release of antigen over time and prolonged exposure to the immune system. However, four subsequent lines of evidence indicate that a depot effect is likely not important for the adjuvant effect of alum. First, after intramuscular injection, most of the antigen diffuses away from the injection site within hours of administration (4). Second, administration of antigen adsorbed to alum does not increase the half-life of antigen *in situ* (2). Third, excision of the injection site within a few hours after vaccine administration did not reduce the magnitude of the ensuing antigen-specific immune responses (5). Finally, Munks et al. demonstrated that alum induces fibrin-dependent nodules at the injection site, but that these nodules do not play a part in the adjuvant effect (6). Taken together, these data strongly rule out any role of antigen depot in alum’s mode of action.

It has long been known that physical interaction of the vaccine antigen with alum is necessary for the full adjuvant effect (1), suggesting that alum functions, at least in part, as a delivery system. This could be accomplished by facilitating co-delivery of the antigen and adjuvant to the appropriate physical location, thereby ensuring that the inflammatory response to alum is directed toward the co-administered antigen. Indeed, alum induces local inflammation at the injection site, irrespective of whether antigen has been adsorbed (7) and the enhancement of antigen-specific immunity is often lost if the antigen and alum are administered at separate locations (8). Particulate vaccine formulations generally are more readily internalized by antigen-presenting cells (APCs) than are soluble antigens and the same is true for alum-adsorbed antigens. The mechanism by which antigen uptake is facilitated is not yet clear, but a recent study suggested that this may occur in the absence of uptake of alum by APCs. Crystalline alum was shown to bind lipids on the surface of APCs and trigger a cellular activation cascade leading to initiation of an immune response, but without itself being internalized by the cells (9), suggesting an indirect role in delivering antigen into the antigen processing pathway. These results are in contrast with a previous study using confocal microscopy showing that alum was internalized by APCs (10). In addition, alum crystals can be found in the endosomes of blood cells using electron microscopy (Latz, personal communication).

The innate immune system is a complex network of sensing pathways that function to rapidly alert the host to infections, cancers, and cellular dysfunction. In the context of vaccines, it has become clear that signaling the innate immune system is an important early aspect in the development of an effective antigen-specific immune response and is one of the key roles for a vaccine adjuvant. *In vitro* studies have shown that alum can facilitate activation of DCs, as measured by increased surface expression of co-stimulatory molecules CD80 and CD86, and secretion of cytokines (11). It is not known whether this is the result of direct cellular signaling and a molecular target, if one exists, has not yet been identified. Injection of vaccines containing alum elicits profound broad local effects on the immune system. Within a few hours after injection, pro-inflammatory cytokines are released and there is an influx of inflammatory monocytes followed by dendritic cells (DCs), natural killer (NK) cells, neutrophils, and eosinophils by 24 h (12, 13). During this time, a constellation of genes are up-regulated, including those encoding cytokines and chemokines (7) which may function to facilitate the recruitment and activation of APCs at the site of injection. These APCs may then internalize vaccine antigens and migrate to the draining lymph node to prime lymphocytes (14).

The molecular mechanisms involved in the response to alum are being elucidated, but more than one pathway may be involved and there are some conflicting results. Unlike the immune stimulatory properties of TLR agonists, which require the adaptor molecules MyD88 and TRIF, the adjuvant effects of alum are not impaired in the absence of these proteins (15), suggesting that alum does not signal in a TLR-dependent fashion. Several studies performed *in vitro* on mouse and human cells have demonstrated that alum can activate the Nlrp3 inflammasome complex, which is required for the processing of several key pro-inflammatory cytokines including IL1. The molecular mechanism of activation of Nlrp3 is not clear, however one report has shown that alum after internalization destabilizes the endosome releasing proteases that are required for Nlrp3 inflammasome activation and IL1 release (10). Consistent with these *in vitro* studies, others have shown Nlrp3 to be required for alum adjuvanticity in mice (16, 17). However, studies performed in other laboratories using different antigens and immunization protocols demonstrated that in some cases Nlrp3 may not be implicated [(18–20)]. Indirect effects of alum can be induced via the release of certain molecules by cells, which then can elicit subsequent adjuvant activity. For example, alum stimulates the induction of uric acid (12), which is produced normally as a damage-associated molecular pattern (DAMP) by injured cells. Released uric acid is then internalized by and activates APCs via the inflammasome, thereby providing a downstream, secondary immunostimulatory signal in response to immunization with alum-containing vaccines. In a similar manner, alum stimulates the release of dsDNA from dying cells and this DAMP seems to play a role in adjuvant activity by promoting antigen presentation to helper T cells (20, 21). In summary, the immunostimulatory effects of alum are broad, rapid, and seem to involve multiple pathways, both direct and indirect. More investigation will be required to fully elucidate these pathways.

## Mode of Acton of Oil-in-Water Emulsions

Oil-in-water emulsions are licensed for use in human influenza vaccines. These include MF59, which was originally licensed in 1997 for influenza vaccines for the elderly, and AS03, which like MF59 was recently approved for pandemic influenza vaccines. MF59 consists of uniform particles ∼160 nm in size generated by microfluidics technology and its main constituents are the naturally occurring oil squalene and the non-ionic surfactants Tween 80 and Span 85. There is a large human clinical experience with MF59, with almost 100 million doses administered over the past 15 years, demonstrating that the adjuvant is safe, well tolerated, effective at increasing vaccine potency, able to reduce the dose of antigen required, and elicits broad-based immunity (22).

Like alum, MF59 was initially thought to exert its adjuvant effect by the formation of an antigen depot. However, studies conducted with labeled MF59 have shown that the adjuvant is quickly drained from the injection site, that only ∼10% of the adjuvant remains at the injection site 6 h after intramuscular administration (23), and that the presence of MF59 does not influence the distribution or the half-life of the co-administered antigen (24). In addition, unlike alum, the adjuvant effects of MF59 can be maintained even when the antigen alone is administered up to 24 h after injection of MF59 at the same site (23). Taken together, these data are not consistent with the hypothesis that MF59 acts as an antigen depot, rather MF59 appears to create an “immunocompetent environment” within the muscle that could facilitate the development of antigen-specific immune responses.

Subsequent work has suggested that MF59 can function as an antigen delivery system, albeit in an indirect fashion. Studies conducted on cells *in vitro* demonstrated that MF59 increased phagocytosis and pinocytosis, and promoted antigen uptake by APCs (25). In that study, neither monocyte-derived DCs (Mo-DCs) nor myeloid DCs (mDCs) isolated from human blood were directly activated by MF59. Rather, MF59 stimulated monocytes, macrophages, and granulocytes to produce the chemokines CCL2, CXCL8, CCL3, and CCL4. In addition, stimulated monocytes underwent phenotypic changes in accordance with their differentiation toward DCs. These data suggested that MF59 does not directly target DCs to internalize antigen, but may act upstream by inducing recruitment of DC precursors and their subsequent differentiation (25).

*In vivo* studies have shown that fluorescently labeled MF59 was found to be co-localized together with the co-administered antigen in immature DCs (DEC205+ MHCII+) infiltrating the mouse muscle at 48 h after injection There was a strong influx of mononuclear cells to the injection site, with a significant proportion of the cells identified as macrophages (F4/80-positive cells) and a minor population of DCs (CD11c-positive cells). This cellular influx induced by MF59 was significantly impaired in CCR2^-/-^ knockout mice, suggesting that MF59 triggers cell recruitment events, at least partially mediated by CCR2, that are required for adjuvanticity(25). In agreement with this hypothesis, microarray analysis demonstrated that MF59 activates the expression of genes encoding cytokines (IL-1b, IL-2), chemokines (Ccl2, Ccl4, Ccl5, Ccl12, Ccl10), and adhesion molecules in the mouse muscle. MF59 also induced the up-regulation of genes coding for Ccr2 and its ligands (7). Furthermore, MF59 promoted a more rapid influx of CD11b+ cells in the muscle compared to other adjuvants (such as alum and CpG oligonucleotides). Some of the genes up-regulated rapidly after MF59 administration were used as biomarkers to identify MF59 target cells. Confocal microscope analysis showed that two of these biomarkers, JunB and Pentraxin 3, were up-regulated in muscle fibers following MF59 treatment, demonstrating that muscle cells are a target of MF59 *in vivo* (7). A subsequent study in mice by Calabro et al. characterized in detail the kinetics and phenotype of the immune cells recruited by MF59 to the injection site (26). Infiltration of granulocytes, such as neutrophils and eosinophils, and potential APCs, such as monocytes, macrophages, and DCs were observed. MF59 was found to be a much stronger activator of cell recruitment than alum and promoted a more efficient uptake of vaccine antigen at injection site. In addition, MF59 significantly increased the number of antigen-loaded APCs in draining LNs compared to alum or non-adjuvanted vaccine (26).

In a recent study, the effects of TLR-independent (alum and MF59) and TLR-dependent (R848, CpG, and Pam3CSK4) adjuvants were characterized using DNA microarray *in vitro* and *in vivo* (27). The transcription profiles from adjuvant-treated cells *in vitro* and injected mouse muscles and their draining lymph nodes (LN) *in vivo* were quite different for the two different adjuvant classes. In contrast to TLR agonists, MF59 and alum did not modulate transcription of cytokine mRNAs by splenocytes *in vitro*. After intramuscular injection, MF59-induced a localized immunostimulatory environment in the muscle but did not modulate the transcriptome in the draining LN and did not induce any antigen-independent activation of B and T cells. In contrast, some of the TLR agonists (such as R848) elicited effects distant from the injection site and modulated gene transcription in LNs in an antigen-independent matter leading to polyclonal T and B cell activation. Finally, immune responses enhanced by MF59 to tetanus and influenza antigens were found to be independent of the presence of interferon type I, unlike R848 which displayed dependency on this cytokine (27).

It has been proposed that adjuvanticity of some particulate adjuvants (including alum) depends on the activation of a protein complex called the Nlrp3 inflammasome that processes certain pro-inflammatory cytokines like pro-IL1β through Caspase 1 (12, 16). Two independent studies have demonstrated that MF59-induced adjuvant effects are independent of Nlrp3 and Caspase 1 (19, 28). However, it was shown that the effects of MF59 depend on the apoptosis-associated speck-like protein containing CARD (ASC), which is a common adaptor of inflammasome complexes (28). Hence, it is possible that ASC might also have an inflammasome-independent function or that inflammasomes different from Nlrp3 might play a role. Experiments conducted using mice deficient in innate immune pathways have shown that enhancement of immune responses to a recombinant meningococcus B vaccine by MF59 required the adaptor molecule MyD88 (19). Yet, MF59 has not been shown to be an agonist of any of the TLR that depend on MyD88 for signaling. Possible explanations include that MF59 induces the release of endogenous TLR agonists at the injection site or that MF59 targets other MyD88-dependent pathways involving the receptors for IL1 family cytokines (IL1R, IL18R, IL33R) or the TACI receptor. As is the case for alum, further studies are required to better understand the mode of action of MF59.

AS03 is another squalene-based emulsion, but differs from MF59 in the absence of the Span85 surfactant and, more importantly, in the presence of α-tocopherol. These differences in the formulation markedly affect the biological activity of the emulsions, mainly due to the immunostimulatory activity of α-tocopherol. Unlike MF59, which activates innate immunity only locally at the injection site, AS03 triggers innate immune responses in the injected muscle and in the draining LN of immunized mice. This activation of the lymph node is independent of the antigen but depends on the presence of α-tocopherol (29).

## Mode of Action of Toll-Like Receptor Agonists

In addition to alum and oil-in-water emulsions, which have been used extensively in human vaccines, various other adjuvants have been evaluated in human clinical trials (see Table 1). Many of these experimental adjuvants are known to target elements of innate immune signaling pathways, in particular the TLRs but also Nod-like receptors, RIG-I-like receptors, and C-type lectin receptors. These PRRs function to provide a first line of immune defense against incoming pathogens by interacting with molecular signatures commonly found in microbes but not in host cells (so called pathogen associated molecular patterns or PAMPs). Examples include, but are not limited to, dsRNA and ssRNA from viruses, CpG motifs from bacterial DNA, certain lipids, lipopeptides and glycans from bacterial cell wall components, flagellin from bacteria, zymosan from yeast, and profilin from protozoa. The importance of the innate immune system in potentiating the adaptive immune response is well established and the critical role this signaling plays in adjuvant function is becoming appreciated. It is likely that the potency of vaccines based on whole organisms is due, at least in part, to stimulation of TLRs. For example, the Yellow fever vaccine, which is based on an attenuated live virus, has been shown to interact with at least four TLRs (30). For this reason, agonists of TLRs and other PRRs are attractive targets as vaccine adjuvants. Following is a brief summary of the key aspects of the TLR agonists that have been achieved proof of concept in humans.

TLR4 is a cell surface PRR that recognizes several PAMPs, including lipopolysaccharides (LPS) from bacteria, and is the target for the well-established adjuvant MPL. Normally, LPS is toxic and not appropriate for use in human vaccines. However, MPL is based on the TLR4-active element of LPS from Salmonella and its toxicity is ∼1000-fold lower than LPS. MPL is an active and safe component of licensed vaccines against hepatitis B and HPV (see Table 1), and more than 100,000 human doses have been administered (31). This TLR4 agonist is typically used in combination with alum and as a consequence enhances both protective antibody responses, as well as promoting a Th1-type of helper T cell response (32). Preclinical and clinical evaluation of MPL and MPL-like synthetic analogs has demonstrated its broad utility as a vaccine adjuvant in animal models of infectious (33, 34) and non-infectious diseases, including allergy (35) and cancer (36).

TLR9 is an endosomal PRR that recognizes DNA with certain motifs containing unmethylated CpG residues more often found in microbial than eukaryotic DNA. Adjuvants directed toward this TLR are perhaps the best studied and most complex of the TLR agonists. For example, there are various types of these CpG motifs, all of which are dependent upon TLR9 but have different qualitative and quantitative effects on the immune response (37) In addition, CpG motifs exhibit species-specific differences (38) that have complicated development of this class of adjuvants. Nevertheless, TLR9 agonists are being evaluated in the later stages of clinical development for infectious disease and allergy indications. For example, a commercial hepatitis B virus (HBV) vaccine formulated with CpG enhanced vaccine potency in humans, as measured by higher levels of protective antibodies with more rapid kinetics and with fewer immunizations than the vaccine alone (39). Although the currently licensed HBV vaccines are very effective, a major limitation is that certain individuals (∼5–10% of the general population depending on geography) do not respond to vaccination even after multiple administrations. The addition of CpG to the vaccine reduces the proportion of these non-responders (40), demonstrating that adjuvants may provide a solution to this limitation. CpG can be effective as a vaccine adjuvant by simple mixing with antigen, but increased potency and lower requirements for antigen dose can be achieved by conjugation of CpG directly to antigen. This approach has been particularly useful for modulation of immune responses to allergens and human trials are underway as a potential therapeutic intervention for treatment of allergic responses (41).

TLR5 is a cell surface PRR that recognizes a particular bacterial protein called flagellin. Because this TLR agonist is proteinaceous in nature, it offers the possibility of creating recombinant fusion proteins containing both an antigen and adjuvant. This approach has been shown to be effective in animal models for influenza using a fusion between flagellin and the hemagglutinin protein. Early human clinical trials have demonstrated proof of concept for the safety and utility of this strategy (42), and opens the possibility of exploring the use of other protein-based TLR agonists such as zymosan and profilin. One potential pitfall of this methodology is the uncertain effects on structural integrity and preservation of important B cell epitopes in the antigen.

TLR7 and 8 are related PRRs found in the endosomes of various immune cells and function to recognize certain ssRNA molecules rich in uridine residues, as is found in viral RNA. Interaction with these TLRs can be mimicked using synthetic compounds, such as imidazoquinolines and the guanosine analog Loxoribine (43). TLR7 activation by the imidazoquinoline imiquimod is an effective topical treatment approved for human use against HPV-induced genital warts and basal cell carcinoma. Imiquimod and a potent related molecule resiquimod have been shown to function as vaccine adjuvants enhancing both antibody and T cell responses in various models including non-human primates (44). Some human vaccine clinical trials have been conducted using topical application of TLR7 agonists at the vaccine injection site, but so far there has been no observed adjuvant effect (45).

TLR3 is an endosomal PRR that recognizes dsRNA, such as is produced during cytoplasmic viral replication. Poly(I:C), which is composed of a mixture of dsRNA species varying considerably in size, has been demonstrated to be an effective vaccine adjuvant in various animal models and for cancer immunotherapy (46). A synthetic dsRNA of defined size and sequence is under development for use as an adjuvant for an mRNA-based vaccine. This two component RNA vaccine (mRNA to mediate antigen expression *in situ* and non-coding dsRNA to stimulate the innate immune system via TLR3) is efficacious in animal models of influenza and cancer (47), and has been shown to be safe and immunogenic as a cancer vaccine strategy in humans (48).

## Summary

The beneficial effects of vaccine adjuvants can be manifest in various ways, including (1) increasing vaccine potency to attain higher levels of immunogenicity and protective efficacy (e.g., alum for various viral and bacterial vaccines), (2) reducing the dose of antigen required for effectiveness (e.g., MF59 for influenza vaccines), (3) increasing the speed and reducing the number of immunizations required to achieve effectiveness (e.g., AS04 for hepatitis B vaccine), (4) broadening the repertoire of antibody responses (e.g., MF59 for influenza vaccines), and (5) modulating the phenotype of T cell responses. Adjuvants have been in use for these purposes for most of the past century, but until relatively recently adjuvant development has been predominated by empiricism. However, our growing insight into innate immune signaling pathways and the key roles PRRs play in the recognition of microbial signatures provides an opportunity to take rational approaches in the design and optimization of new vaccine adjuvants (as demonstrated in the preceding section). Knowledge of the molecular target (e.g., a specific TLR) enables vaccine developers to harness the power of drug discovery tools, such as (1) high throughput screening to mine large libraries of small molecular compounds for a particular property or activity, (2) medicinal chemistry to design and synthesize families of related compounds, and (3) computational approaches to elucidate structure activity relationships and aid in the optimization of adjuvant candidates (49). However, this process of optimization will create strong pro-inflammatory molecules, hence it will be important to strike the correct balance between potency and safety. To this end, these small molecule immune potentiators can be designed not only to maximize beneficial immunologic effects, but also to minimize undesirable side effects by (1) manipulating pharmacokinetic properties that affect biodistribution of the compounds (e.g., limit systemic exposure) and (2) facilitating interaction with formulations designed to ensure localized co-delivery of the antigen and immunostimulatory compound. This balance will be further influenced by the relative risks versus benefits of including an adjuvant in a vaccine. For example, the tolerance for added risk of side effects by inclusion of an adjuvant in a vaccine will be very different for prevention of a low likelihood event in healthy people (e.g., anthrax exposure) compared to treatment of an ongoing life-threatening condition (e.g., cancer). The breadth of molecular targets for small molecule compounds coupled with the diversity of disease targets and patient populations for vaccines has created a fertile area for novel adjuvant discovery and development.

## Conflict of Interest Statement

The authors are employees of Novartis Vaccines and Diagnostics.
